# Arginine Vasopressin Deficiency and Oxytocin Deficiency in the Endocrine Clinic

**DOI:** 10.1210/clinem/dgaf651

**Published:** 2025-12-09

**Authors:** Cihan Atila, Julie Refardt, Mirjam Christ-Crain

**Affiliations:** Department of Endocrinology, Diabetology and Metabolism, University Hospital Basel, Basel 4031, Switzerland; Department of Clinical Research, University of Basel, University Hospital Basel, Basel 4031, Switzerland; Department of Endocrinology, Diabetology and Metabolism, University Hospital Basel, Basel 4031, Switzerland; Department of Clinical Research, University of Basel, University Hospital Basel, Basel 4031, Switzerland; Department of Internal Medicine, Section of Endocrinology, Erasmus Medical Center, Rotterdam 3015, the Netherlands; Department of Endocrinology, Diabetology and Metabolism, University Hospital Basel, Basel 4031, Switzerland; Department of Clinical Research, University of Basel, University Hospital Basel, Basel 4031, Switzerland

**Keywords:** polyuria polydipsia, diabetes insipidus, copeptin, primary polydipsia, posterior pituitary, diagnostic, hyponatremia, hypernatremia, desmopressin, treatment

## Abstract

The hypothalamo-neurohypophysial system consists of specialized neurons in the supraoptic and paraventricular nuclei that project to the central nervous system and the posterior pituitary, where they secrete arginine vasopressin (AVP) and oxytocin (OXT) into the systemic circulation.

AVP is the key endocrine regulator of water balance via V2 receptor–mediated aquaporin-2 insertion in renal collecting ducts and modulates vascular tone via V1 receptors. OXT plays a central role in labor and lactation, but also influences metabolism, social behavior, emotional processing, and stress regulation. AVP deficiency (formerly central diabetes insipidus) results from hypothalamic-posterior pituitary disruptions or injury due to surgery, trauma, tumors, infiltrative or autoimmune disease, vascular events, or genetic causes. It is characterized by hypotonic polyuria, polydipsia, and dehydration risk, and is diagnosed by distinguishing it from AVP resistance and primary polydipsia, with copeptin-based tests providing high diagnostic accuracy. Treatment relies on desmopressin and careful education to prevent both dehydration and hyponatremia.

In contrast, OXT deficiency has only recently been recognized as a potential clinical entity, particularly in patients with hypothalamic-pituitary disruptions or injury and concurrent AVP deficiency. Emerging evidence links it to social dysfunction, anxiety, and reduced quality of life. Diagnosis remains challenging due to unreliable basal OXT levels and limited stimulation tests; novel approaches, including 3,4-methylenedioxymethamphetamine (MDMA) challenge and neurophysin I as a surrogate marker, are under investigation. Preliminary studies suggest intranasal OXT may improve socioemotional outcomes, but robust evidence from randomized controlled trials is needed.

The hypothalamo-neurohypophysial system comprises magnocellular neurons in the supraoptic (SON) and paraventricular (PVN) nuclei, whose axons project via the infundibulum to the posterior pituitary (1-4). These neurons synthesize arginine vasopressin (AVP) and oxytocin (OXT) with their carrier neurophysins, releasing both hormones into the systemic circulation at neurovascular junctions, as well as centrally within the hypothalamus and other brain regions (Fig. 1) (5, 6). AVP is the principal endocrine regulator of water homeostasis through V2 receptor-mediated aquaporin-2 insertion in the renal collecting duct, and it also contributes to vascular tone via V1a receptors and stress responses via V1b in pituitary corticotrope cells (3, 7). OXT drives labor and lactation, supports metabolic regulation, and exerts central effects on social behavior, trust, empathy, stress regulation, and emotional processing (see Fig. 1) (8-11).

**Figure 1.**
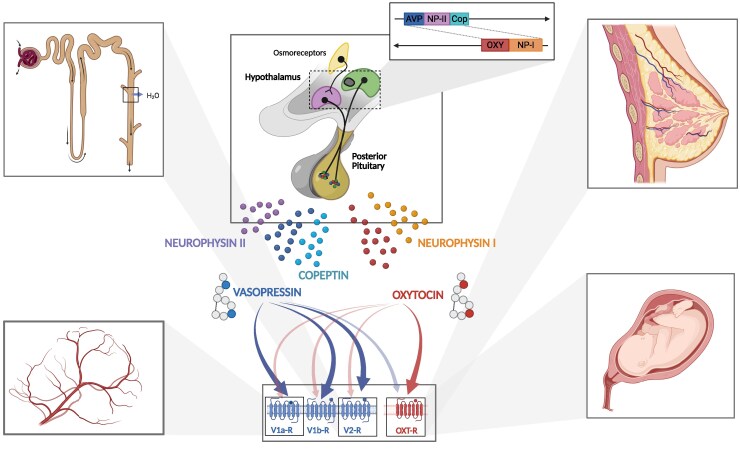
The hypothalamic-posterior-pituitary axis. Magnocellular neurons of the supraoptic and paraventricular nuclei project to the posterior pituitary, where arginine vasopressin (AVP) and oxytocin (OXT) are released together with their carrier proteins copeptin/neurophysin II and neurophysin I, respectively. AVP exerts its effects via V1a, V1b, and V2 receptors, regulating renal water reabsorption (V2R, left upper panel), vascular tone (V1aR, left lower panel), and stress responses via anterior pituitary corticotrope cells (V1bR). OXT acts through the OXT receptor (OXTR) to mediate uterine contractions during parturition (right lower panel) and milk ejection during lactation (right upper panel). Figure created with biorender.com.

AVP deficiency (formerly central diabetes insipidus) typically arises from hypothalamic-posterior pituitary disruptions or injury due to neurosurgery, trauma, tumors, infiltrative disease, autoimmunity, vascular events, or genetic defects (12-14). It manifests clinically with hypotonic polyuria, compensatory polydipsia, and risk of dehydration if water access is impaired. Diagnosis requires confirmation of hypotonic polyuria and distinction from AVP resistance (formerly nephrogenic diabetes insipidus) and primary polydipsia, with copeptin-based testing offering high diagnostic accuracy (14-16). Management involves desmopressin replacement and patient education to prevent both dehydration and hyponatremia, alongside treatment of underlying causes (17, 18).

While AVP deficiency is well-characterized, OXT deficiency has only recently emerged as a clinically relevant entity, particularly in patients with hypothalamic or pituitary damage and concurrent AVP deficiency (19, 20). Potential manifestations include socioemotional dysfunction, anxiety, and reduced quality of life (QoL) (12, 20, 21). Diagnosis is challenging because basal OXT levels are unreliable and standard provocation tests provide limited weak stimulation; stronger stimuli (eg, MDMA) and surrogate markers such as neurophysin I are under investigation (20, 22). Preliminary reports suggest intranasal OXT may improve social behavior and emotion recognition in hypothalamic disorders, but evidence from randomized controlled trials of other entities (eg, obesity or autism spectrum disorder) remains inconclusive, and trials in patients with hypothalamic disorders are lacking (21, 23-25).

This review summarizes current knowledge on the pathophysiology, diagnostic approaches, and therapeutic strategies for AVP deficiency and OXT deficiency, with a focus on their relevance in hypothalamic-pituitary disorders.

## Arginine Vasopressin Deficiency

To avoid (fatal) complications due to confusion with diabetes mellitus, the new name AVP deficiency, instead of “central diabetes insipidus,” was introduced in 2023 (26-30). The updated terminology is based on pathophysiological accuracy, referring to the impaired AVP synthesis or secretion. AVP resistance replaces “nephrogenic diabetes insipidus” and describes renal insensitivity to AVP action. Importantly, the renaming also creates a clearer framework for discussing co-occurring posterior pituitary hormone deficiencies, particularly OXT deficiency, which may accompany AVP deficiency in the context of hypothalamic-pituitary disorders and have distinct clinical consequences. This nomenclature change is a patient-driven, safety-focused shift toward diagnostic clarity and improved clinical care (26).

In addition to AVP deficiency and AVP resistance, primary polydipsia can also lead to hypotonic polyuria and polydipsia (31-34). Despite their similar clinical presentation, their underlying mechanisms, clinical trajectories, and management differ significantly. AVP deficiency results from impaired synthesis or secretion of AVP by the hypothalamus or posterior pituitary (35-37). This deficiency leads to the inability of the kidneys to concentrate urine, causing the excretion of large volumes of hypotonic urine and triggering thirst to maintain fluid balance (38, 39). Causes of AVP deficiency are listed in Table 1. AVP resistance, on the other hand, is characterized by normal or elevated AVP levels but a renal insensitivity to its action (40-42). Causes of AVP resistance include inherited mutations in the *AVPR2* or *AQP2* genes, chronic lithium use, hypercalcemia, hypokalemia, and certain nephrotoxic medications (43, 44). Primary polydipsia, in contrast, originates from excessive water intake rather than a defect in the AVP axis (32, 34, 45). Once thought to occur predominantly in psychiatric conditions such as schizophrenia, primary polydipsia is now also recognized in individuals with hypothalamic dysfunction or in behavioral contexts, for instance, health-conscious individuals who habitually consume large amounts of water. Chronic excessive fluid intake suppresses endogenous AVP release and reduces medullary solute gradient, gradually impairing the kidney's concentrating capacity (46). Over time, this can mimic the biochemical picture of AVP deficiency or resistance with impaired urinary concentration capacity, especially if the underlying cause of polydipsia is not recognized.

**Table 1.** Causes of arginine vasopressin deficiency

**Post-surgical or post-traumatic**	Neurosurgery Head trauma (deceleration injury) Radiotherapy
**Pituitary or hypothalamic tumors**	Craniopharyngioma Meningioma Germinoma Rathke cleft cyst Pituitary adenoma Astrocytoma
**Idiopathic**	
**Infiltrative**	Neurosarcoidosis Histiocytic disorder
**Inflammatory/autoimmune lesions**	Neuroinfundibular lymphocytic hypophysitis Granulomatous hypophysitis Xanthomatous hypophysitis IgG4-related hypophysitis
**Genetic/familial**	AVP-Neurophysin-II gene mutations
**Pituitary apoplexy**	Sheehan syndrome
**Anatomic variations**	Empty sella
**Metastatic lesions**	Lymphoma Breast cancer Lung cancer
**Infectious**	Meningitis Encephalitis Tuberculosis

Abbreviations: AVP, arginine vasopressin; IgG4, immunoglobulin G4.

### Cause of Arginine Vasopressin Deficiency

The causes of AVP deficiency are heterogeneous and can be broadly categorized into acquired, genetic, and idiopathic forms (see Table 1) (12, 37, 47-49). There are 3 principal mechanisms underlying acquired forms:

1. Anatomical destruction of vasopressinergic neurons, often by neoplasms such as craniopharyngiomas, germinomas, metastases, or infiltrative diseases (eg, Langerhans cell histiocytosis, sarcoidosis).
2. Traumatic injury to the hypothalamic-neurohypophyseal axis, either due to traumatic brain injury (TBI) or neurosurgical intervention, especially transsphenoidal or suprasellar procedures.
3. Autoimmune destruction, frequently in the setting of lymphocytic hypophysitis, sometimes accompanied by anterior pituitary hormone deficiencies.

Genetic forms of AVP deficiency, most commonly autosomal dominant mutations in the *AVP-neurophysin-II* gene, typically present in early adulthood and result in intracellular accumulation of AVP precursors, causing progressive dysfunction (50, 51). Idiopathic cases were historically reported in up to 50% of cases (26, 52-56). However, with improved diagnostic procedures, many such cases are now recognized as autoimmune in origin (26, 52-56). In a recent series, less than 10% to 30% remained classified as idiopathic (26, 52).

#### Special considerations: AVP deficiency due to traumatic injury

Postsurgical AVP deficiency is the most common etiology and typically occurs 1 to 2 days after pituitary surgery, resolving within 2 to 5 days in most cases (57, 58). A triphasic response may occasionally occur with first transient AVP deficiency, followed by hyponatremia from unregulated AVP release, and finally (in some cases) persistent AVP deficiency occurring due to neuronal loss (59, 60). TBI and subarachnoid hemorrhage are also well-established causes of traumatic AVP deficiency (61). Transient AVP deficiency occurs in approximately 20% of moderate-to-severe TBI cases and in 15% of nontraumatic subarachnoid bleeds (62). Pituitary adenomas, by contrast, rarely cause AVP deficiency preoperatively. The presence of AVP deficiency in a patient with a sellar mass should prompt consideration of diagnoses such as craniopharyngioma, malignancy, or granulomatous disease (57).

#### Special considerations: adipsic AVP deficiency

In most cases of AVP deficiency, the thirst mechanism is preserved, and compensatory polydipsia prevents hypernatremia (18, 63, 64). In rare cases, damage to the anterior hypothalamus involving osmoreceptors localized in the organum vasculosum of the lamina terminalis in the anterior wall of the third ventricle, and in the subfornical organ, leads to adipsic AVP deficiency, in which both thirst sensation and AVP release are impaired (65-67). This life-threatening condition is most often seen after extensive surgery for suprasellar tumors. Adipsic AVP deficiency carries high morbidity and mortality and requires careful fluid management with often fixed fluid intake, as patients lack behavioral compensation for free water loss (68).

#### Special considerations: gestational AVP deficiency

Placental trophoblasts produce vasopressinase, detectable from week 10, with levels rising up to 300-fold by the third trimester and falling to baseline within 2 weeks post partum (69). Production is proportional to placental mass, with twin/multiple pregnancies showing higher levels, and accelerates AVP degradation. AVP deficiency during pregnancy can result in or manifest as (1) worsening of a preexisting condition with partial, asymptomatic disease that may become symptomatic due to inability to compensate for vasopressinase activity; symptoms often recur in subsequent pregnancies; or (2) a pregnancy-induced condition typically in the late second or early third trimester from peak vasopressinase activity (69-74).

### Diagnosis of Arginine Vasopressin Deficiency

**Figure 2.**
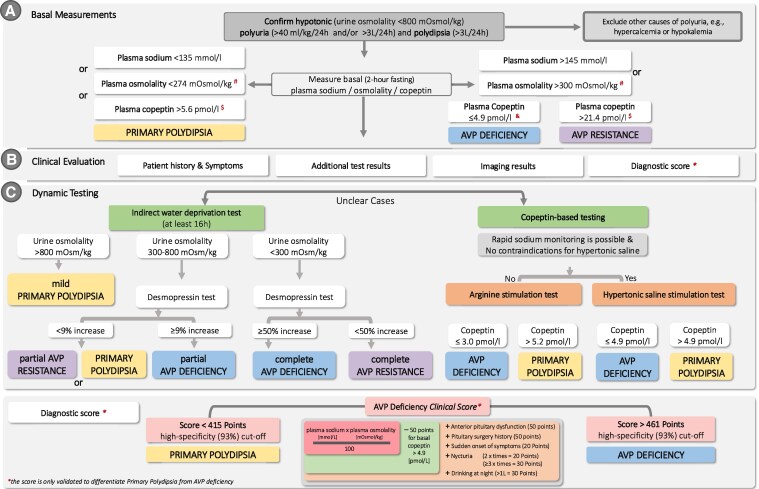
Diagnostic algorithm in arginine vasopressin deficiency. A, The evaluation begins with confirmation of hypotonic polyuria and exclusion of alternative causes, followed by basal measurements of plasma sodium, osmolality, and copeptin. In addition, certain clinical features are suggestive of AVP deficiency, including frequent nocturia (>2-3 times/night), sudden onset of polyuria/polydipsia, high nocturnal fluid intake (>1 L/night), and a history of anterior pituitary dysfunction or pituitary surgery. B, Several complementary sources of information are integrated. Clinical history and symptomatology are assessed, including nocturia, onset pattern, thirst behavior, and prior pituitary disease. When available, additional anterior pituitary hormone profiles and structural or functional magnetic resonance imaging findings (eg, loss of posterior pituitary bright spot, hypothalamic-pituitary abnormalities) are incorporated. In addition, a validated point-based diagnostic score can be applied to distinguish AVP deficiency from primary polydipsia without the need for dynamic testing. C, Uncertain cases are further assessed using dynamic tests. In the indirect water deprivation test, AVP activity is assessed indirectly by the capacity to concentrate urine. Persistently low urine osmolality (<300 mOsm/kg) suggests AVP deficiency or resistance. Following desmopressin, urine osmolality rises more than 50% (119) in complete AVP deficiency but remains below this threshold in AVP resistance. Patients with partial AVP deficiency or primary polydipsia typically achieve urine osmolality of 300 to 800 mOsm/kg; an additional desmopressin-induced increase of 9% or more indicates partial AVP deficiency, whereas a decrease of less than 9% supports primary polydipsia. Copeptin-based testing (unstimulated or stimulated by arginine or hypertonic saline) provides an alternative pathway (see text for details). *The score is validated only to differentiate primary polydipsia from AVP deficiency (for the full score, see original publication (85)). $To differentiate between primary polydipsia (basal copeptin >5.6 pmol/L) and AVP resistance (basal copeptin >21.4 pmol/L), overlapping copeptin values may occur in the presence of confounding factors such as nausea or distress. Therefore, basal copeptin levels should only be interpreted in the absence of severe nausea or physical/psychological stress. &Basal copeptin of 4.9 pmol/L or less for AVP deficiency can be used only if concomitant plasma sodium is 147 mmol/L or greater. # Plasma osmolality should be interpreted with certainty only if plasma glucose and plasma urea are within the normal range.

Hypotonic polyuria-polydipsia syndrome presents a diagnostic challenge across multiple specialties. The clinical presentation is marked by excessive urine output (hypotonic polyuria defined as >40 mL/kg body weight/24 hours with urine osmolality <800 mOsm/kg) and fluid intake (polydipsia defined as excessive fluid intake of >3 L/day) can arise from several distinct pathophysiological processes (Fig. 2) (14, 16, 17, 31, 37). The main challenge is to differentiate between AVP deficiency, AVP resistance, and primary polydipsia.

Accurate diagnosis relies on a comprehensive clinical history, biochemical evaluation, and functional testing. Plasma sodium, plasma and urine osmolality, urine volume, and 24-hour fluid intake are essential first steps. A detailed medication history and evaluation for hypercalcemia or hypokalemia are important, particularly when AVP resistance is suspected. Other causes of polyuria must be excluded, for example, osmotic diuresis in uncontrolled diabetes mellitus and postobstructive diuresis.

#### Baseline clinical and laboratory assessment

Over the last decade, copeptin has proven to be a reliable and stable surrogate marker for AVP (15, 75, 76). Copeptin is the C-terminal part of the precursor peptide pre-pro-vasopressin (see Fig. 1). Unlike AVP, which is unstable and technically challenging to measure, copeptin is highly stable in plasma and serum and can be quantified with commercially available assays (77). Two CE-(Conformité Européenne)-certified assays currently in clinical use are the automated immunofluorescent assay (KRYPTOR platform) and the manual sandwich immunoluminometric assay (78). In contrast, noncertified enzyme-linked immunosorbent assays have shown poor correlation and are not approved for clinical diagnostic purposes (78). Basal copeptin samples must be collected under controlled, stress-free conditions to avoid misinterpretation (77, 79).

Already in the first studies assessing copeptin as a diagnostic marker in the differential diagnosis of polyuria-polydipsia syndrome, it was shown that an elevated copeptin level greater than 21.4 pmol/L reliably diagnoses AVP resistance (see Fig. 2) (80, 81). However, the distinction between AVP deficiency and primary polydipsia requires a more detailed work-up. In the 2 largest prospective studies, the degree of polyuria and polydipsia showed substantial overlap between the 2 entities (81-83). Psychiatric disorders, particularly depression, have historically been reported more frequently in patients with primary polydipsia; however, more recent data indicate a comparable prevalence of psychiatric conditions (∼30%) in both groups (26, 82, 84).

An analysis of 299 patients from 2 international multicenter studies, including patients with AVP deficiency and primary polydipsia undergoing hypertonic saline testing, assessed the diagnostic utility of basal laboratory parameters and developed a score integrating laboratory values, clinical symptoms, and medical history (see Fig. 2; for details, see original publication) (85). Data were obtained at the initial consultation, including basal laboratory measurements. The analysis showed that a basal plasma sodium concentration greater than 145 mmol/L identified AVP deficiency with 100% specificity, while sodium less than 135 mmol/L or copeptin greater than 5.6 pmol/L identified primary polydipsia with 100% specificity (85). In addition, certain clinical features were highly suggestive of AVP deficiency, including frequent nocturia (>2-3 times/night), sudden onset of polyuria/polydipsia, high nocturnal fluid intake (>1 L/night), and a history of anterior pituitary dysfunction or pituitary surgery. A simple point-based score incorporating these basal laboratory values, symptoms, and medical history achieved a diagnostic accuracy of 86% (area under the curve = 91%) for identifying AVP deficiency, enabling diagnosis in many cases without the need for further dynamic testing (see Fig. 2) (85).

#### Indirect water deprivation test

In patients with unclear baseline laboratory and clinical investigations, dynamic testing is required. For decades, the indirect water deprivation test has served as the method for differentiating the main causes of polyuria-polydipsia syndrome (86-88). The test operates by indirectly assessing endogenous AVP activity through a prolonged period of water restriction, followed by evaluation of the renal concentrating response to exogenous desmopressin (see Fig. 2). However, despite its widespread use, the diagnostic accuracy of the indirect water deprivation test is limited, particularly in borderline cases such as partial AVP deficiency and primary polydipsia. This stems in part from the aforementioned reduced AVP secretion and renal medullary concentration gradient in chronic polydipsia, but also compensatory upregulation of V2-receptors in some patients with AVP deficiency, which may artificially enhance urine concentrating capacity. Overall, the indirect water deprivation test achieves a diagnostic accuracy of only 70% to 77% and is therefore no longer considered the diagnostic gold standard (80, 81, 89-91).

#### Copeptin on hypertonic saline stimulation

Among the available stimulation tests, the hypertonic saline test has demonstrated the highest diagnostic accuracy for distinguishing between AVP deficiency and primary polydipsia. In this protocol, 3% hypertonic saline is administered as a 250-mL bolus over 15 minutes, followed by a body-weight–adapted continuous infusion (80-82, 92). The goal is to elevate plasma sodium to at least 147 mmol/L or greater, which reliably stimulates copeptin secretion. To guarantee the safety of the test, sodium levels must be measured at least every 30 minutes using rapid methods (eg, blood gas analysis). Once the target sodium threshold is reached, a blood sample for copeptin measurement is obtained, and the infusion is terminated. Immediate oral and parenteral rehydration is essential to normalize sodium levels safely. Using a copeptin cutoff of more than 4.9 pmol/L, patients with primary polydipsia can be accurately differentiated from those with AVP deficiency. Two large prospective studies confirmed a diagnostic accuracy of 96% to 97%, with sensitivity and specificity of 91% to 93% and 99% to 100%, respectively (81, 82). The test should be performed only in settings equipped for rapid sodium monitoring, where constant supervision can be provided during the test. It is contraindicated in patients with heart failure, epilepsy, older individuals (limited safety in >65 years old), and children. Patients with known adrenal insufficiency require stress dose corticosteroid coverage.

#### Copeptin on arginine stimulation

Arginine, a known stimulus of anterior pituitary hormone secretion, has been investigated as an alternative means of stimulating copeptin release. In a prospective study of 96 patients with polyuria-polydipsia syndrome and healthy controls, an infusion of arginine (0.5 g/kg body weight diluted in 500 mL of normal saline over 30 minutes) led to a significant rise in copeptin levels (83). In patients with polyuria-polydipsia syndrome, a first single-center study showed a high diagnostic accuracy using a copeptin cutoff of 3.8 pmol/L or greater measured 60 minutes after infusion (83). However, a recent prospective multicenter diagnostic trial could not confirm this high diagnostic accuracy for a single cutoff. Instead, levels of 3.0 pmol/L or less or greater than 5.2 pmol/L reliably identified more than half of patients with AVP deficiency and primary polydipsia, respectively, with a specificity exceeding 90% (82, 93). The most common adverse effect of arginine infusion was mild nausea, reported by approximately 50% of participants (82). Compared with hypertonic saline, arginine stimulation is better tolerated, requires no continuous laboratory monitoring, and can be completed within 1 hour, making it a feasible alternative in routine clinical practice. Nevertheless, its diagnostic accuracy remains inferior to that of the hypertonic saline test (82).

Given the findings of the hypertonic and arginine stimulation tests, a practical diagnostic algorithm is recommended (see Fig. 2). While many patients can be diagnosed by basal sodium and copeptin measurement as well as medical history, hypertonic saline-stimulated copeptin testing remains the gold standard when safety monitoring and rapid sodium testing are available. Arginine stimulation offers a simple, well-tolerated alternative when applying low and high copeptin cutoffs for diagnostic classification.

#### Emerging copeptin-based tests

Alternative agents to hypertonic saline and arginine infusion have been explored. Macimorelin, an oral ghrelin receptor agonist approved for growth hormone testing, showed no effect on copeptin levels (94, 95). More promising, however, is glucagon: In a recent study, 1 mg subcutaneous glucagon stimulated copeptin release in healthy controls and patients with primary polydipsia, but not in those with AVP deficiency (96). A copeptin cutoff of greater than 4.6 pmol/L at 150 minutes post injection yielded 100% sensitivity and 90% specificity. More recently, in a novel diagnostic test using a single oral dose of urea (0.5 g/kg), copeptin remained undetectable in AVP deficiency but rose significantly in primary polydipsia (97). A cutoff of 3.5 pmol/L at 120 minutes achieved 93% sensitivity and specificity. In addition, mannitol infusion was shown to stimulate copeptin in healthy volunteers, with results in patients with AVP deficiency and primary polydipsia pending.

#### Magnetic resonance imaging findings in arginine vasopressin deficiency

When AVP deficiency is confirmed, magnetic resonance imaging (MRI) of the hypothalamic-pituitary region has to be performed. The so-called pituitary bright spot (a hyperintense area in the posterior pituitary lobe, thought to reflect stored AVP in neurosecretory granules) was initially described as pathognomonic for AVP deficiency (98-101). However, larger studies have demonstrated an age-related absence of the bright spot in more than half of healthy individuals, as well as in some patients with congenital AVP resistance (102, 103). Moreover, cases of AVP deficiency with a persistent bright spot have been reported. Recent analyses found the bright spot to be absent in up to 70% of patients with AVP deficiency, but also in up to 39% of patients with primary polydipsia (81, 82). Consequently, the presence or absence of the bright spot should not be used as the sole diagnostic criterion. A thickened pituitary stalk is likewise not pathognomonic. When the diagnosis remains uncertain, dynamic functional testing to distinguish the subtype of polyuria-polydipsia syndrome may be required.

### Management of Arginine Vasopressin Deficiency

Desmopressin (1-deamino-8-D-arginine vasopressin), a synthetic AVP receptor agonist, is the mainstay in the treatment of AVP deficiency (104, 105). AVP has both V1a (vasoconstrictive) and V2 (antidiuretic) receptor activity, with a short plasma half-life (∼10 minutes) (38). In contrast, desmopressin is a synthetic analogue with a deaminated cysteine and D-arginine substitution, conferring high selectivity for V2 receptors, negligible V1a activity, and a markedly prolonged half-life (6-14 hours depending on route) (106-108). These modifications reduce pressor effects, extend dosing intervals, and improve safety in long-term therapy for AVP deficiency. Therapeutically, desmopressin dosing is individualized (104, 109, 110). To avoid complications such as fluid retention or hyponatremia, it is recommended to titrate desmopressin beginning with a nighttime dosage (eg, desmopressin tablets starting dose 0.05-0.1 mg once daily, typical total 0.1-0.8 mg/day divided into 1 to multiple doses); intravenous desmopressin (eg, 1-4 mcg/day divided) is used acutely (Table 2) (109).

**Table 2.** Desmopressin dosages according to different formulations

Formulation	Concentration	Starting dosage, µg
IV/SC/IM	4 µg/mL	1
Intranasal	0.1 mg/mL (10 µg/dose)	10
By mouth	100 µg or 200 µg tablets	50-100
Sublingual	60 µg/120 µg/240 µg tablets	60

Abbreviations: IM, intramuscular; IV, intravenous; SC, subcutaneous.

#### Prevention of desmopressin-induced hyponatremia

Desmopressin carries a well-recognized risk of dilutional hyponatremia (106, 111, 112). This complication arises from desmopressin's sustained antidiuretic action, which is not subject to the rapid feedback inhibition by hypoosmolality. Even modest fluid intake under desmopressin can lead to substantial water retention and subsequent hyponatremia. Retrospective and survey-based studies suggest that 22% to 31% of patients experience mild to moderate hyponatremia during long-term desmopressin therapy, and up to 26% may require hospitalization due to hyponatremia-related complications (63, 112-115). Early symptoms often go unrecognized and include nausea, vomiting, headaches, and lethargy, progressing to confusion, seizures, or even coma in severe cases.

**Figure 3.**
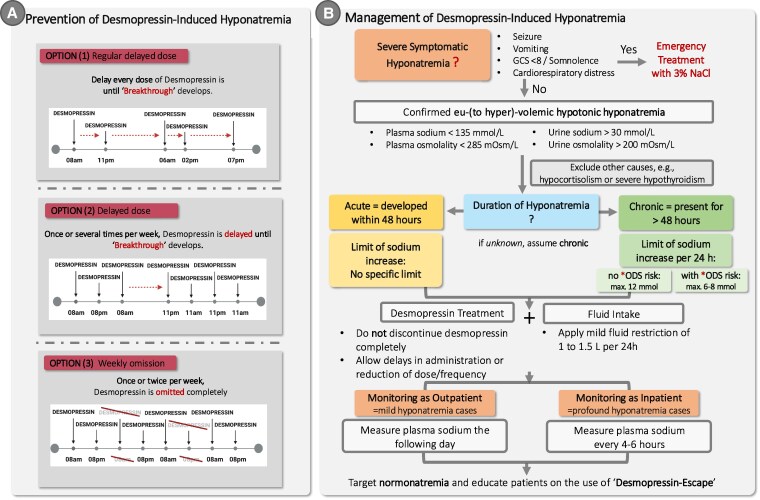
Prevention and management of desmopressin-induced hyponatremia. A, Left panel: Prevention strategy using the desmopressin-escape method, in which desmopressin doses are routinely delayed or omitted until polyuria (“breakthrough”) occurs, thereby reducing hyponatremia risk. Patients are instructed to take the next dose only after breakthrough symptoms (eg, frequent voiding of dilute urine, strong thirst) appear. Options include weekly omission, delayed dosing, or regular delayed dosing. B, Right panel: Management algorithm for confirmed euvolemic to hypervolemic hypotonic hyponatremia under desmopressin therapy. After excluding other causes, patients are assessed for the severity and chronicity of hyponatremia. Emergency treatment with 3% NaCl is indicated in severe symptomatic cases. Generally, desmopressin can be continued, dose adjustments are considered, and mild fluid restriction is applied. Sodium correction should not exceed 6 to 8 mmol/L per 24 hours in patients with ODS risk and should not exceed 12 mmol/L per 24 hours in patients without ODS risk. Monitoring frequency depends on severity, with inpatients requiring sodium checks every 4 to 6 hours. ODS, osmotic demyelination syndrome. *Known ODS risk factors: profound hyponatremia of less than 110 mmol/L, alcohol use disorder, liver disease, malnutrition, hypokalemia, and hypophosphatemia.

To address this, the desmopressin-escape method has been proposed and increasingly recommended as a safe and pragmatic approach for long-term management of AVP deficiency (Fig. 3) (26). This strategy involves withholding or delaying a dose of desmopressin at regular intervals, commonly once or twice per week, to permit spontaneous aquaresis and restore water balance. During this “escape” period, the desmopressin effect wears off, allowing the patient to excrete any retained free water through transient polyuria. This physiological break reduces the risk of cumulative water retention and subsequent hyponatremia. Recent data support the effectiveness of the desmopressin-escape method. Patients who regularly performed this method had significantly lower reported episodes of outpatient hyponatremia and hospitalization for hyponatremia, compared to those who were either unaware of the method or did not implement it despite awareness (26).

There is some debate over whether formulation affects risk. Postmarketing surveillance suggested a higher hyponatremia risk with nasal desmopressin formulations, with reports indicating a 60% risk reduction on switching to oral preparations (114, 116). However, more recent analyses have failed to show a significant difference in hyponatremia prevalence between formulations, and most patients switching from nasal to oral do so for reasons of convenience and symptom control rather than biochemical risk.

#### Management of desmopressin-induced hyponatremia

Despite this recognized complication, guidance on how to manage desmopressin-induced hyponatremia remains scarce in clinical guidelines, and mismanagement can lead to devastating neurological consequences (117). A case series of 15 patients with symptomatic desmopressin-induced hyponatremia illustrates the clinical pitfalls and treatment outcomes of 2 distinct management strategies (111). Among 13 patients in whom desmopressin was discontinued on recognition of hyponatremia, the combination of drug withdrawal and hypertonic saline administration led to free water diuresis, resulting in a mean serum sodium correction of 37 mmol/L over 48 hours. This overcorrection was associated with severe neurological injury in 12 patients, including 3 fatal cases and permanent cognitive or vegetative impairment in 9 patients. In contrast, 2 patients managed with continued desmopressin administration alongside controlled correction with hypertonic saline (mean sodium correction of 11 mmol/L over 48 hours) recovered without neurological sequelae. These findings support the hypothesis that withholding desmopressin in patients with symptomatic hyponatremia can precipitate a sudden shift from an antidiuretic to a diuretic state, leading to uncontrolled sodium correction and heightened risk of osmotic demyelination syndrome (ODS) (see Fig. 3) (118).

The resulting clinical recommendations are therefore the following:

1. Do not withhold desmopressin in the acute management phase, even in the setting of hyponatremia. Discontinuation may trigger abrupt water diuresis and rapid correction of sodium, increasing the risk for ODS.
2. Initiate mild fluid restriction. In severe symptomatic hyponatremia, hypertonic saline therapy should be applied to reverse cerebral edema (eg, 100-150 mL bolus of 3% saline, followed by further correction based on clinical and biochemical response).
3. Monitor serum sodium closely (every 2-4 hours) during correction. Avoid sodium correction exceeding 12 mmol/L in 24 hours in patients without ODS risk and 6 to 8 mmol/L in 24 hours in patients with ODS risk.

#### Management of AVP deficiency during pregnancy

During pregnancy, the pituitary enlarges, especially the posterior lobe, lowering the osmotic threshold for AVP release and thirst. This results in approximately 5 mmol/L lower serum sodium and approximately 10 mOsm/kg lower osmolality. Desmopressin is safe in pregnancy and lactation, unaffected by vasopressinase, and does not induce labor (74). However, increased desmopressin dosages might be needed due to the degradation of the remaining AVP through vasopressinase in patients with partial AVP deficiency. In newly diagnosed conditions, patients should start with the lowest effective bedtime dose (eg, 10-µg intranasal or 0.05 mg tablets), titrating by symptoms and sodium (target 135-140 mmol/L). After delivery, the desmopressin dosage often has to be decreased (69, 70, 73).

#### Management and prevention of hypernatremia

Even without desmopressin, patients with intact osmoregulated thirst perception and unrestricted access to water are generally able to compensate for urinary water losses through increased fluid intake. Consequently, hypernatremia rarely occurs in patients. However, limited fluid availability or excessive extrarenal water loss (eg, restricted intake, vomiting, diarrhea, impaired consciousness, or acute intercurrent illness) can rapidly precipitate life-threatening dehydration (26, 29, 117). Supporting this, Behan et al (63) reported high hypernatremia rates of approximately 20% in hospitalized patients, likely reflecting insufficient awareness of appropriate fluid management and treatment errors by health-care providers. Notably, 1 in 4 hospitalized patients in our study reported dehydration symptoms due to the inability to take desmopressin while fasting (26). Such situations have been associated with serious adverse outcomes, including fatalities. Several studies underscore the urgent need for improved education of medical personnel and the designation of desmopressin as a high-alert medication with 24-hour hospital availability (117). When dehydration occurs, total body water deficit can be estimated using the formula: 0.6 × premorbid weight × (1 − 140/[measured Na⁺ in mmol/L]) (31). The goal is to achieve a positive water balance equal to approximately 50% of the calculated free water deficit (31, 119). Hypotonic fluids should be used, preferably administered enterally; if intravenous therapy is required, 5% dextrose is recommended (alternatively, 0.45% sodium chloride) (119, 120). In acute hypernatremia developing over a few hours, rapid correction of up to 1 mmol/L per hour is safe and improves prognosis (119). In chronic or unknown-duration hypernatremia, correction should proceed more slowly (≤0.5 mmol/L per hour) to prevent cerebral edema and seizures (121, 122). Overall, a targeted reduction of about 10 mmol/L per day, aiming for a serum sodium of 145 mmol/L, is recommended (119, 120).

**Figure 4.**
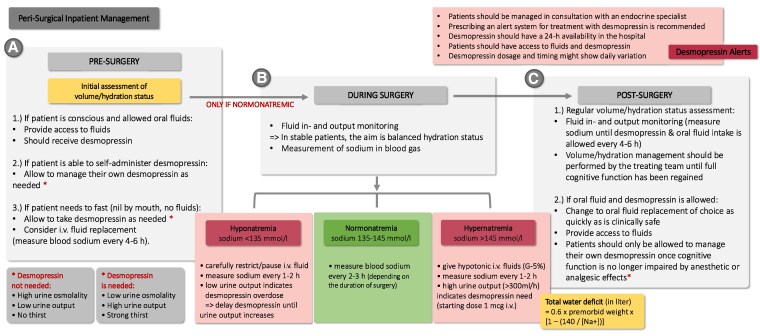
Recommended perisurgical inpatient management. A, Pre surgery: If patients are conscious and allowed oral fluids, access to fluids and desmopressin should be ensured. Patients able to self-administer should manage desmopressin themselves. During fasting, desmopressin may be given as needed, with intravenous (i.v.) fluid replacement considered and plasma sodium monitored every 4 to 6 hours. B, During surgery: An initial assessment of volume and hydration status should be performed. Monitoring of fluid input/output and sodium (blood gas) is required, with the aim of maintaining balanced fluid status in stable patients. Hyponatremia (sodium <135 mmol/L) requires i.v. fluid restriction/pausing and frequent sodium checks (1-2 hours), with delayed desmopressin if urine output is low. Normonatremia (135-145 mmol/L) requires regular sodium monitoring (2-3 hours). Hypernatremia (>145 mmol/L) is managed with hypotonic i.v. fluids, frequent sodium checks (1-2 hours), and desmopressin initiation if urine output is high (>300 mL/h). C, Post surgery: Regular assessment of hydration, input/output, and plasma sodium is mandatory until oral fluids and desmopressin are resumed. Once allowed, oral fluid replacement should be prioritized, with fluids placed within easy reach. Patients may self-administer desmopressin as needed. Additional recommendations include close consultation with endocrinology, 24-hour hospital availability of desmopressin, and use of treatment alerts.

A proposed perisurgical inpatient management procedure is presented in Fig. 4. A particularly vulnerable subgroup comprises patients with adipsic AVP deficiency (65, 66). Management requires close monitoring of serum sodium and patient education on hydration self-assessment. Standard practice involves prescribing a fixed daily fluid intake (adjusted according to body-weight changes) in combination with a fixed desmopressin dose (14, 67). In these patients, they and their caregivers should both be instructed to seek prompt medical evaluation during situations carrying an increased risk of hyponatremia or hypernatremia.

## Oxytocin Deficiency

### Anatomy and Physiology of Oxytocin Secretion

OXT is a conserved nonapeptide hormone that plays a pivotal role in labor, lactation, and the modulation of social and emotional behaviors (8). It is closely related to AVP, differing by only 2 amino acids (123, 124). OXT concentrations in the brain are up to 1000-fold higher than in plasma, with a central half-life (∼19 minutes) more than 3 times longer than in the periphery (∼6 minutes) (20, 38, 125, 126). OXT biosynthesis begins with the translation of pre-pro-OXT, which is processed to pro-OXT and cleaved into the active nonapeptide and its carrier protein, neurophysin I (NP-I) (see Fig. 1) (123). NP-I is essential for proper folding, axonal transport, and storage (125, 127, 128). The OXT receptor (OXTR) is widely expressed in the brain, including the hypothalamus, amygdala, cingulate cortex, olfactory system, and limbic areas, as well as peripherally in reproductive tissues (uterus, ovaries, testes, mammary glands) and nonreproductive organs (kidneys, pancreas, adipose tissue, adrenal glands) (3, 129).

During late pregnancy, uterine sensitivity to OXT increases markedly due to OXTR upregulation of up to 200-fold in the myometrium (130, 131). This facilitates rhythmic uterine contractions that contribute to cervical dilation and fetal expulsion. Additionally, OXT promotes prostaglandin release from the decidua, enhancing myometrial contractility (131). In lactation, OXT is involved in the milk-ejection reflex (130). Infant suckling triggers pulsatile OXT release from the posterior pituitary, which in turn stimulates milk let-down (130). Beyond reproductive tissues, OXT exerts pleiotropic effects via systemic circulation. In the ovary and testis, OXT influences steroidogenesis and facilitates ovulation and ejaculation (132-134). In ovarian granulosa cells, OXT receptor activation enhances progesterone synthesis through upregulation of steroidogenic enzymes and bone morphogenetic protein-15 signaling, with minimal effect on estradiol production and inhibiting luteinizing hormone (135-137). In prostate cancer cell lines, OXT differentially modulates key androgen-synthesizing enzymes (3β-hydroxysteroid dehydrogenase [HSD3B] and 5α-reductase) in a cell line–dependent manner, increasing HSD3B activity in PC-3 (androgen-independent prostate cancer) cells but inhibiting it in LNCaP (androgen-sensitive prostate cancer) cells (138). In the cardiovascular system, it contributes to natriuresis and vasodilation, and has been implicated in modulating atherosclerotic processes through its anti-inflammatory and antioxidative effects (139-142). Intranasal OXT has been shown to improve glucose tolerance and β-cell responsiveness, reduce hunger-driven and reward-driven food intake, and limit snack consumption both in lean and obese individuals (10, 143-145). In the skeletal system, OXTRs are expressed both on osteoblasts and osteoclasts. OXT promotes bone formation, enhances ossification, and inhibits bone resorption (20, 146). Clinically, low OXT levels have been associated with reduced bone mineral density in patients with AVP deficiency (147, 148).

Centrally, OXT is a key neuromodulator of social, emotional, and stress-related behaviors. Released from hypothalamic neurons via dendritic and axonal pathways, it acts on OXTRs throughout the brain. OXT facilitates maternal care, pair bonding, social recognition, and empathy (149-152). It enhances the salience of social cues and promotes the formation of social memory, thereby supporting prosocial behaviors such as trust, cooperation, and intimacy (152, 153). In animal models, central OXT administration increases partner preference, reduces aggression, and enhances affiliative behavior (154-156). Similar effects have been observed in humans, in whom intranasal OXT improves emotion recognition, trust, and interpersonal connectedness (157-160). Sex-dependent effects have been reported in specific brain regions such as the anteroventral periventricular nucleus (161). OXT also exerts potent anxiolytic and antistress effects. It dampens amygdala reactivity while increasing insular activation in response to emotional stimuli and modulates the hypothalamic-pituitary-adrenal axis to lower cortisol responses. OXT has been shown to buffer social stress, particularly when combined with social support (162). For example, participants receiving both OXT and emotional support exhibited the lowest cortisol responses to stress (163).

### Symptoms Associated With Oxytocin Deficiency

#### Changes in psychosocial, emotional, and eating behavior

Hypopituitarism (HPD) is a condition with a prevalence of 21 to 42 cases per million, characterized by partial or complete deficiency of one or more pituitary hormones (164). While anterior pituitary hormone deficiencies are well recognized and posterior pituitary AVP deficiency is effectively managed with desmopressin, OXT deficiency has only recently gained attention. Given the anatomical proximity of vasopressinergic and oxytocinergic neurons, damage causing AVP deficiency likely affects OXT pathways as well.

Patients with HPD frequently experience comorbidities such as sexual dysfunction, obesity, osteoporosis, and neuropsychiatric symptoms, including anxiety, depression, alexithymia, and social withdrawal, that are not fully explained by anterior pituitary hormone deficiencies alone (84, 165). Recent data suggest that a state of OXT deficiency may contribute to these clinical features, particularly in patients with hypothalamic damage (20, 21). This includes individuals with suprasellar tumors (eg, craniopharyngiomas, germinomas), congenital malformations, infiltrative diseases, TBI, or those who have undergone neurosurgery or radiotherapy. Damage to OXT-producing neurons may contribute to emotional dysregulation, social impairments, and altered eating behavior. In clinical studies, OXT deficiency has been linked to impaired social cognition, reduced empathy, insecure attachment, social anxiety, diminished trust, and alexithymia (166-168). Emotionally, individuals may exhibit heightened stress sensitivity, depressive symptoms, and reduced resilience to psychosocial stressors. Metabolically, OXT deficiency may lead to hyperphagia and obesity, particularly hypothalamic obesity following PVN damage (10). OXT also interacts with mesolimbic reward pathways, and low OXT levels are associated with altered hedonic responses to food (169). In this context, OXT deficiency has been implicated both in restrictive eating (eg, anorexia nervosa) and hyperphagic obesity (169). More than half of patients treated for sellar tumors develop hypothalamic obesity, which is associated with reduced quality of life, metabolic syndrome, and increased mortality (170). The extent of hypothalamic damage and the presence of AVP deficiency are predictors of this condition (171). Craniopharyngioma, in particular, is known to damage both AVP and OXT neurons. Longitudinal studies report persistent alterations in personality, social functioning, and emotion regulation, despite adequate desmopressin treatment (170, 172). In the largest survey of patients with AVP deficiency, 64% reported impaired QoL, including difficulties in social engagement, recreation, and emotional well-being. Psychological complaints were common: A total of 25% reported anxiety, 25% sleep disturbances, 23% depressed mood, and 12% personality changes (26). Although AVP-deficient patients are at the highest risk for concurrent OXT deficiency, not all individuals seem to be affected equally. Milder cases may preserve or regenerate OXT neurons. Nonetheless, elevated adrenocorticotropin and cortisol responses to stress in patients with AVP deficiency suggest impaired hypothalamic-pituitary-adrenal axis regulation, potentially due in part to coexisting OXT deficiency (173).

#### Changes and complications in obstetric functions

In the context of OXT deficiency, especially in patients with HPD or AVP deficiency, one might expect obstetric complications due to impaired myometrial contractility and inadequate stimulation of breast myoepithelial cells (130, 174). Post partum, insufficient OXT release may impair uterine involution and elevate the risk of hemorrhage due to suboptimal uterine contractions and could impair the milk ejection reflex, potentially leading to breastfeeding failure. However, despite these mechanistic expectations, current clinical data remain limited. Case series in patients with HPD or AVP deficiency have not consistently demonstrated a need for exogenous OXT to initiate or sustain labor (70, 175). Some patients undergo spontaneous labor without OXT administration, and approximately half of the patients are able to breastfeed at discharge. These findings suggest that pituitary-derived OXT may be partially preserved or that compensatory mechanisms can support parturition and lactation. In support of this, mice lacking OXT or OXTR exhibit normal parturition and delivery of pups, indicating that OXT signaling is not essential for the initiation or completion of labor (176, 177). However, these knockout mice are unable to lactate and cannot eject milk in response to suckling, resulting in neonatal death unless milk ejection is pharmacologically restored (178, 179).

### Diagnosis of Oxytocin Deficiency

**Figure 5.**
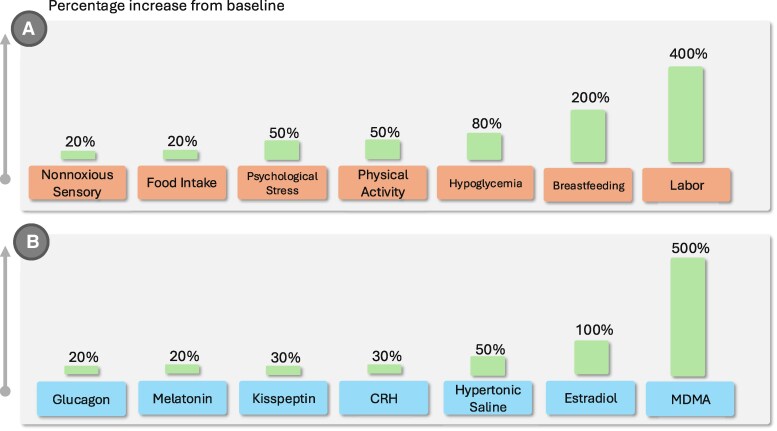
Selected physiological and exogenous stimuli of peripheral oxytocin release. A, Upper panel: Physiological and behavioral stimuli, including nonnoxious sensory stimulation (∼20%) (203, 204), food intake (∼20%) (205), psychological stress (∼50%) (204, 206, 207), physical activity (∼50%) (167, 204), hypoglycemia (∼80%) (208), breastfeeding (∼200%) (130, 204), and labor (∼400%) (131). B, Lower panel: Exogenous pharmacological and endocrine stimuli, including glucagon (∼20%) (187), melatonin (∼20%) (185), kisspeptin (∼30%) (209), corticotropin-releasing hormone (CRH, ∼30%) (186), hypertonic saline (∼50%) (192), estradiol (∼100%) (125, 210), and 3,4-methylenedioxymethamphetamine (MDMA, ∼500%) (196). Values represent approximate increases relative to baseline, as reported studies show variability between publications.

Despite its physiological and behavioral relevance, no validated clinical test currently exists to diagnose OXT deficiency in routine practice. Peripheral OXT can be measured in plasma, saliva, or urine, but these assays are hampered by substantial technical limitations, including poor specificity, high variability, and inconsistent correlation with central OXT activity (180-184). Consequently, single-point basal OXT measurements are unreliable (180). Early studies examining basal OXT levels in patients with hypothalamic-pituitary disorders have yielded conflicting results (166-168, 185-189). Some found no difference in basal saliva OXT levels between craniopharyngioma patients and healthy controls, whereas others reported lower basal OXT in patients with hypothalamic-pituitary disorders, regardless of AVP deficiency, and associated these levels with impaired social-emotional processing, such as facial emotion recognition. In male patients with AVP deficiency, symptoms of depression, anxiety, and alexithymia were more pronounced (189). When OXT levels were pooled over time, a slight reduction was seen in patients (189). In contrast, other studies reported elevated basal OXT in patient groups, underscoring the lack of reliability of basal measurements. These discrepancies can be explained by the pulsatile secretion of OXT, short half-life, and lack of standardization in assay techniques (180, 190). Central and peripheral OXT release may also be dissociated. Notably, central and peripheral OXT levels correlate only after stimulation, not at baseline, suggesting that a provocative test is required, similar to testing strategies used for adrenal insufficiency or growth hormone deficiency (191). To date, common stimuli such as corticotropin-releasing hormone, arginine, hypertonic saline, macimorelin, glucagon, or melatonin have failed to reliably stimulate OXT secretion; meanwhile, older data showed an estradiol-induced increase (Fig. 5) (185-187, 192-195). A test must ideally both induce supraphysiological OXT release and elicit central behavioral responses to be diagnostically meaningful (191). A recent study used 3,4-methylenedioxymethamphetamine (MDMA) as a pharmacological provocation agent (196, 197). A single 100-mg dose of MDMA led to an 8-fold increase in plasma OXT in healthy individuals, but elicited no OXT increase in patients with AVP deficiency (197). Correspondingly, controls experienced characteristic psychoactive effects such as empathy, trust, openness, and reduced anxiety, whereas patients displayed blunted or absent responses, both emotionally and physiologically. Notably, fear recognition in the Facial Emotion Recognition Task was reduced in healthy participants but unchanged in patients, pointing toward central OXT dysfunction. However, MDMA is not viable for routine use due to cardiovascular side effects and regulatory restrictions. Lower, safer doses or pharmacological alternatives mimicking MDMA's effects on OXT release and social cognition are being explored.

Another key challenge in diagnosing OXT deficiency lies in the lack of a robust biomarker. Traditional OXT immunoassays are time-consuming (16-24 hours), prone to nonspecific binding, and require complex extraction. An emerging solution is the use of NP-I, the carrier protein coreleased with OXT, as a surrogate biomarker (22, 198, 199). NP-I is secreted in equimolar concentrations with OXT but has a longer half-life, making it less susceptible to rapid degradation and moment-to-moment fluctuations (125, 128). Recent studies indicate that NP-I strongly correlates with OXT (*R* = 0.92) and with subjective psychosocial responses such as trust and reduced fear (22, 198). Under MDMA stimulation, healthy individuals showed a 20-fold increase in NP-I, compared to minimal changes in patients. NP-I also offers superior analytical performance: No extraction is needed, and assay time is reduced to less than 2 hours. This is analogous to the use of copeptin as a surrogate marker for AVP, suggesting that NP-I could fill a similar role for OXT. Further validation studies are needed to establish diagnostic cutoffs.

### Oxytocin Replacement Therapy

OXT replacement therapy is not currently part of standard clinical practice for patients with HPD (20, 21). Although there is growing recognition that coexisting OXT deficiency may contribute to the broader psychosocial, emotional, and metabolic burden observed in patients, clinical guidelines do not currently recommend OXT replacement due to a lack of data underlining a benefit of OXT replacement therapy (20, 21). For neurobehavioral symptoms, the role of chronic OXT deficiency has become a topic of increasing research interest (20). Nonetheless, evidence supporting OXT replacement therapy remains limited and preliminary. A systematic review of the OXT system in patients with craniopharyngioma identified only 2 case reports and 1 small pilot study (200-202). One case described a young girl with panhypopituitarism and hypothalamic damage who showed improved social engagement after receiving 4 IU/day intranasal OXT. Another involved a 13-year-old boy with hypothalamic obesity who was treated with 6 IU/day OXT for 10 weeks, then combined with naltrexone for 38 weeks. This regimen led to reductions in body mass index and hyperphagia. In a cross-sectional pilot study of 10 craniopharyngioma patients (9 with AVP deficiency), a single 24-IU dose of intranasal OXT acutely improved recognition of negative facial emotions (200). However, the small sample size and absence of a placebo control limit the generalizability of these findings. Generally, evidence from randomized controlled trials of other entities (eg, obesity or autism spectrum disorder) remain inconclusive regarding the beneficial effects of intranasal OXT (23-25). Recognizing the unmet need, 3 randomized, placebo-controlled clinical trials are currently under way to investigate intranasal OXT as a novel treatment for psychological and socioemotional symptoms in patients (NCT06789705, NCT06676774, NCT06460948, NCT06036004, NCT06808516, NCT04789148).

## Data Availability

No new data were generated or analyzed in this review. Data supporting the discussed findings are available in the cited original studies.

## Abbreviations

AVP: arginine vasopressin
desmopressin: 1-deamino-8-D-arginine vasopressin
HPD: hypopituitarism
HSD3B: 3β-hydroxysteroid dehydrogenase
MDMA: 3,4-methylenedioxymethamphetamine
MRI: magnetic resonance imaging
NP-I: neurophysin I
ODS: osmotic demyelination syndrome
OXT: oxytocin
OXTR: OXT receptor
PVN: paraventricular nuclei
QoL: quality of life
TBI: traumatic brain injury
